# The Intrinsic Virtues of EGCG, an *Extremely Good Cell Guardian*, on Prevention and Treatment of Diabesity Complications

**DOI:** 10.3390/molecules25133061

**Published:** 2020-07-04

**Authors:** Maria Assunta Potenza, Dominga Iacobazzi, Luca Sgarra, Monica Montagnani

**Affiliations:** 1 Department of Biomedical Sciences and Human Oncology, University of Bari “Aldo Moro”, 70124 Bari, Italy; sgarraluca@gmail.com; 2 Faculty of Health Sciences, Bristol Medical School, University of Bristol, Bristol BS8 1QU, UK; domingaiacobazzi@live.it

**Keywords:** epigallocatechin-3-gallate (EGCG), oxidative stress, diabetes, obesity, redox balance

## Abstract

The pandemic proportion of diabesity—a combination of obesity and diabetes—sets a worldwide health issue. Experimental and clinical studies have progressively reinforced the pioneering epidemiological observation of an inverse relationship between consumption of polyphenol-rich nutraceutical agents and mortality from cardiovascular and metabolic diseases. With chemical identification of epigallocatechin-3-gallate (EGCG) as the most abundant catechin of green tea, a number of cellular and molecular mechanisms underlying the activities of this unique catechin have been proposed. Favorable effects of EGCG have been initially attributed to its scavenging effects on free radicals, inhibition of ROS-generating mechanisms and upregulation of antioxidant enzymes. Biologic actions of EGCG are concentration-dependent and under certain conditions EGCG may exert pro-oxidant activities, including generation of free radicals. The discovery of 67-kDa laminin as potential EGCG membrane target has broaden the likelihood that EGCG may function not only because of its highly reactive nature, but also via receptor-mediated activation of multiple signaling pathways involved in cell proliferation, angiogenesis and apoptosis. Finally, by acting as epigenetic modulator of DNA methylation and chromatin remodeling, EGCG may alter gene expression and modify miRNA activities. Despite unceasing research providing detailed insights, ECGC composite activities are still not completely understood. This review summarizes the most recent evidence on molecular mechanisms by which EGCG may activate signal transduction pathways, regulate transcription factors or promote epigenetic changes that may contribute to prevent pathologic processes involved in diabesity and its cardiovascular complications.

## 1. Introduction

The term “diabesity” has been coined to emphasize the close relationship between obesity and Type 2 diabetes mellitus (T2DM) [1,2,3]. Obesity is a chronic, multifactorial metabolic disease characterized by abnormal or excessive body fat accumulation, in which energy consumption (food intake) overcomes energy expenditure [4]. The increase in obese or overweight individuals (i.e., body mass index (BMI) ≥ 30 kg/m^2^) worldwide is explained by the progressive adoption of a Western lifestyle, characterized by a combination of excessive food intake and physical inactivity (WHO Diabetes mellitus, 2018. WHO Published by World Health Organization [5]). Currently, the dramatic prevalence of obesity among young people, together with incorrect habits in everyday life such as sedentarism and fatty acids (FAs) overload, has been associated with a parallel increase in the likelihood of developing T2DM [6]. Diabetes, in turn, is a silent and sneaky metabolic derangement where altered homeostasis of carbohydrates and lipids—resulting from impaired insulin signaling pathways—is directly coupled with vascular changes that affect both the morphology and physiology of cardiovascular system and significantly contribute to the overall increased morbidity and mortality risk [7,8,9,10]. Investigations on mechanisms and cellular signaling pathways that lie underneath the impaired cardiovascular function in diabesity have been intensely pursued, with the aim to identify crucial targets for preventative and therapeutic strategies. It has long been accepted that, even before the onset of over hyperglycemia in T2DM, the selective insulin resistance with impaired activity of phosphatidylinositol (PI) 3-kinase (PI3K)/Akt pathway results in deregulation of signaling cascades involved in nitric oxide (NO) production and endothelial protection [11,12]. The very same unbalanced activity in this branch of insulin signaling allows compensatory hyperinsulinemia to overstimulate the unaffected mitogen activated protein (MAP)-kinase pathway, which contributes to trigger endothelial dysfunction by increasing endothelin-1 (ET-1) release, and set a proinflammatory predisposition to pro-thrombotic and pro-atherogenic vascular events [13,14]. Once developed, hyperglycemia profoundly impacts on vascular morphology and physiology through at least four mechanisms: the polyol pathway, the advanced glycation end products (AGEs) formation, the protein kinase C (PKC)-diacylglycerol (DAG) activation and the hexosamine pathways influx [15].

In this scenario, oxidative stress, resulting from the excessive production of reactive oxygen species (ROS) with a concomitant impaired availability of antioxidant defenses, represents the main final common pathway for all the major mechanisms described above [8,16]. ROS derived by the initial overproduction of superoxide by the mitochondrial electron-transport chain contribute to permanent alteration of cell components including proteins and lipids, and may induce post-translational modifications of histones causing chromatin remodeling and change in levels of gene expression [17]. All these phenomena underlie the development and progression of micro-and macrovascular complications, and predispose to cardiovascular morbidity and mortality events [18].

Since oxidative stress is the central self-sustained alteration, whose deranged consequences increase overtime [19], the adoption of “antioxidant” strategies has been repeatedly proposed to counteract damaging mechanisms, limit the injury progression and reduce the most detrimental metabolic and vascular consequences in patients with diabesity. Nevertheless, while preclinical studies have reported that some vitamins and supplements can help lower markers of oxidative stress such as reduced plasma levels of lipoic acid and glutathione -consistently measured in diabetic patients with micro- and–macrovascular complications- other investigations conclude that most of these antioxidants do not accomplish any substantial benefit in humans [8,20,21,22].

These disappointing results have been explained, in part, by the fact that conventional antioxidants usually neutralize reactive oxygen molecules on a one-for-one basis, while diabesity-induced overproduction of superoxide is a continuous process. In more recent years, recognition on the physiological role played by endogenous ROS production has partially changed the paradigm [23]; thus, it has become increasing clear that an effective preventative strategy should display, rather than a generic “antioxidant” activity, the ability to modulate pathways that increase adaptation to stress.

The idea that some nutraceutical compounds may possess more than a simple ROS scavenger activity arises from the observation that some polyphenols, in addition to a general increase in the antioxidant defenses, directly interact with cells in metabolic tissues as well as in the vascular compartment [24]. In line with this, an inverse relationship has been repeatedly observed between dietary polyphenol consumption and risk of cardiovascular morbidity/mortality and diabetes [25,26,27,28]. The reported close association between green tea consumption and decreased risk of cardiometabolic disorders and mortality [29] has highlighted the activity of polyphenolic components contained in different tea preparations [30], suggesting that routinely utilization of some nutraceuticals confers chemopreventive and cytoprotective activities with mechanisms still partially unknown.

The most abundant green tea polyphenol, EGCG, seems implicated in most green tea effects, as proposed by a variety of experimental results from cells, animals or clinical investigations [31,32,33,34,35]. In the context of diabesity, EGCG owns anti-obesogenic properties [36], which span from decrease in body weight [37,38,39], adipose mass [37] or food intake [40], to reduction of triglycerides and cholesterol plasma levels [37,38] and antidiabetic activity that improves glucose metabolism [41,42,43], insulin resistance, hepatic steatosis [37,42] and overall vascular function [44].

In animal models, the improved glycemic profile and the amelioration of insulin resistance mediated by EGCG have been associated with a concomitant decrease in ROS content [45]. However, although EGCG is well renowned for its antioxidant properties [45,46], several additional mechanisms have been proposed overtime [34,47,48,49,50]. Interestingly, the biologic functions of EGCG seem to be concentration-dependent and may vary among a spectrum that ranges from antioxidant to pro-oxidant activities according to the cellular redox context.

In the next paragraphs, epidemiological observations reporting EGCG effects in obesity or diabetes are briefly summarized, with subsequent focus on mechanisms underlying EGCG-mediated protection in lipid alteration, glucose impairment and vascular complications.

## 2. Structure of EGCG: Is an “Antioxidant” or “Pro-oxidant” Molecule?

As previously mentioned, oxidative stress develops when production of free radicals and antioxidant defenses are unbalanced. However, under physiological conditions, small amounts of ROS are produced in the cell, mainly from the mitochondrial transport chain, but also by a number of enzymes localized in the plasma membrane and in the cells cytoplasm [51]. This underlines the role of ROS as fundamental endogenous mediators, whose activity is involved in a variety of signaling cascades and critical for healthy cell function [52,53].

The balance between the oxidative compounds derived from molecular oxygen and the antioxidant defenses in the body gives rise to a dynamic harmony that allows ROS to exert their signaling role without causing collateral damages. This balance can be broken for a variety of reasons, but the extent of damage depends on the nature, the amount of ROS and the place of formation. Under control conditions, endogenous ROS production is carefully regulated to prevent deleterious consequences. The first line of defense against excessive free radicals is a complex enzymatic system that includes superoxide dismutases (SOD), glutathione peroxidases (GPx), thioredoxin reductases (TRXR) and catalases able to convert ROS into less active molecules. The second line of defense involves non-enzymatic antioxidant activity, such as that displayed by nutraceutical substances with a scavenging effect.

As already pointed out, inhibition of ROS by these “pure antioxidants” does not always have a predictable outcome on cell function, mostly because the role of ROS changes under differing environmental conditions. This makes particularly important to identify specific mechanisms for molecules that are expected to target excessive ROS production under pathologic cellular contexts.

The activities of EGCG seem related to its scavenging effect on free radicals [54], but also to inhibition of ROS-generating enzymes, chelation of redox transition-metal ions and upregulation of antioxidant enzymes, thereby contributing with a multiplicity of mechanisms to counteract oxidative stress under diabesity [55]. On top of this, numerous findings from experimental and clinical studies point out to additional properties that seem more directly related to EGCG ability to activate signaling pathways [43]. In this perspective, the unstable chemical structure of EGCG and its ability to spontaneously release ROS (acting as a “pro-oxidant”) may trigger intracellular cascades and reinforce overall effects.

The chemical composition and pharmacokinetic profile of EGCG is extensively described elsewhere [56]. In brief, the polyphenolic structure of EGCG consists of 4 rings, A, B, C and D deriving from the esterification of EGC with gallic acid. A and C rings constitute the benzopyran moiety with a phenyl group at C2 and a gallate group at C3 positions. The B and D rings of EGCG have vicinal 3,4,5-trihydroxy groups, respectively, which confer anti-oxidative potential; in the D ring the galloyl moiety is in the form of an ester at C3, that makes EGCG highly susceptible to nucleophilic attack and favors the formation of strong H-bonds [57]. Furthermore, the B and D rings of EGCG have been associated with an inhibitory effect of proteasome activity in vitro [58], while the A ring seems able to interfere with the heat shock protein 90 [59].

**Figure 1:**
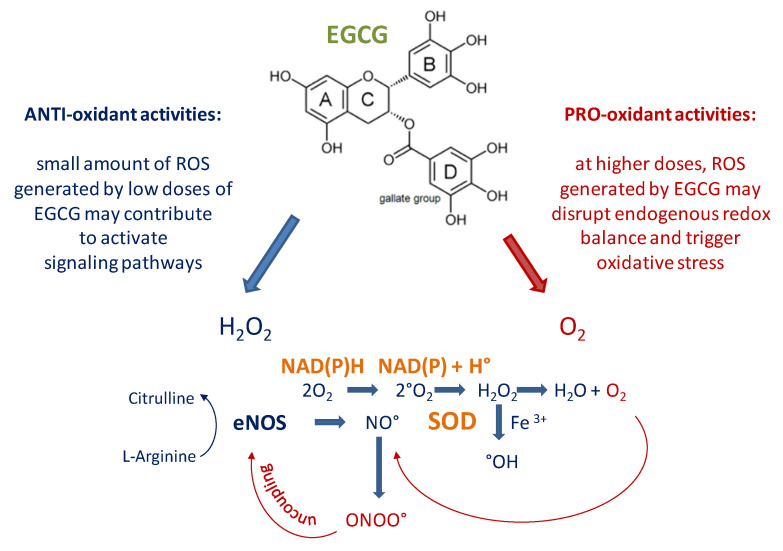
Simplified view of anti- and pro-oxidant activities of epigallocatechin-3-gallate (EGCG). Due to its relative chemical instability, EGCG may undergo autooxidation to produce O_2_ and H_2_O_2_ and accelerate the generation of hydroxyl radical (OH^−^) from Fenton reaction. At lower biologic concentrations (1 μM up to 10 μM) the smaller amounts of intracellular reactive oxygen species (ROS) produced by EGCG are believed to stimulate multiple pathways and promote cellular protective mechanisms. At higher concentrations (>50 μM) the pro-oxidant nature of EGCG predominates and may trigger cytotoxicity by disrupting redox balance mechanisms.

EGCG is relatively unstable and may spontaneously generate free radicals and increase ROS production under certain conditions [60,61,62,63]. For example, EGCG undergoes autooxidation and produces superoxide anion (O_2_) and hydrogen peroxide (H_2_O_2_) with its pyrogallol moiety; moreover, the molecule is known to reduce Fe^3+^ to Fe^2+^ and accelerate the generation of hydroxyl radical (HO^−^), from Fenton reaction [64,65]. Transition metal ions such as Fe^2+^ and Cu^2+^ may therefore contribute to ROS-generating activity of EGCG [66]. The reducing power of EGCG at higher concentrations (>50 μM) may gradually predominate over its ROS scavenging activity and result in the pro-oxidant effect that damages cellular integrity and disrupts nuclear and mitochondrial function. Increased ROS levels by EGCG may in turn decrease the activity of GPx, SOD, catalase and TRXR [67,68,69] (Figure 1).

Recognition of these effects and related cytotoxicity has suggested a significant role of EGCG in cancer therapy, as well as potential negative consequences on healthy cells, particularly in the liver [70,71,72,73,74,75]. However, at lower biologic concentrations (1 μM up to 10 μM) EGCG produces smaller amounts of intracellular ROS that stimulate multiple pathways to promote cellular protective mechanisms, supporting the idea that activation of signaling cascades may, at least in part, depend on EGCG-mediated ROS production [76].

The systemic bioavailability of EGCG in humans after tea consumption is much lower with respect to circulating levels of EGCG after intragastric administration in animals [77,78,79,80]. In part, this is due to EGCG high solubility, which limits its absorption through membranes and in part to its chemical steadiness, that is elevated in an acidic environment such as the stomach during gastric digestion, but decreases rapidly in alkaline duodenal environments [81]. The wide distribution of EGCG in most tissues is explained by its avid binding to albumin, which stabilizes and transports EGCG in the bloodstream [82]. In humans, as in mice, EGCG undergoes similar phase II biotransformation processes that result in methylated, sulfated and glucuronidated products as the major EGCG metabolites [83,84]. Since EGCG cannot be detected in urines, renal excretion seems to be only marginally involved in the rapid fall of its plasma levels once the maximum plasmatic concentration (Cmax) is reached [85]. Instead, as in mice, EGCG may be removed from the bloodstream by the liver and return to the small intestine through the bile [86]. While the entero-hepatic recirculation of EGCG in humans remains to be established, it is likely that this bile-excreted EGCG, if not more extensively degraded, would be degallated by the gut microbiota and then eliminated in urines as EC and EGC metabolites [79].

Thus, pleiotropic effects of EGCG may be related to distinct actions and roles of these potentially bioactive derivatives. Based on pharmacokinetics data, faster and acute effects may be ascribed to direct interaction of EGCG with cells, whereas chronic effects may most likely depend on various EGCG metabolites.

## 3. Effects of EGCG: Evidence from Epidemiological and Intervention Studies

In the ancient Chinese tradition, green tea is said to wash out fat. Beneficial effects of tea and its catechins have been reported in obese individuals [36,87], although discrepancies have emerged based on sex, age or population characteristics: for example, tea consumption was negatively associated with obesity and fasting blood glucose level in women, but not in men, in a Polish population [88], whereas no association was found between green tea consumption and the prevalence of metabolic syndrome or any of its components in a Japanese population [89]. Diverging results may arise from confounding factors related to methods of tea consumption, differences in tea temperature [90] or effects produced by caffeine, simultaneously present in green tea, that synergistically enhances sympathetic nervous system activity and increases energy expenditure by fat oxidation [91].

Interventional studies on effects of green tea for prevention of obesity are not always consistent as well, for dissimilarities in methodological design and protocols or limitations related to gender and number of patients recruited, the degree of obesity or differences in the metabolic asset of insulin-resistant obese patients with respect to subjects overweight, but otherwise healthy [92]. However, a favorable effect of green tea catechins in terms of body weight, BMI, waist circumference, and fat mass reduction has been observed in adults [93,94] and ameliorated fatness, blood pressure and cholesterol levels reported in obese or overweight children [95].

Clinical trials indicate that acute administration of pure EGCG for 2 days has positive effects on metabolic parameters such as respiratory quotient and energy expenditure, decreases lactate concentration in muscles, and may increase fat oxidation thermogenesis as well as a reduction in body weight and body fat in a longer term [96,97,98,99,100].

As far as tea consumption and reduced risk of type 2 diabetes, a positive correlation has been reported in several epidemiological studies [101,102,103,104,105], but not in all subpopulations [106,107]. A meta-analysis including nine cohort studies with follow-up ranging from 5 to 18 years remarks the close relationship between high tea consumption and lower risk of type 2 diabetes development [104], and even a low intake of green tea suggests similar effects on blood glucose [108].

Some intervention studies on diabetic patients report a correlation between high intake of green tea catechins and improved insulin levels or improved blood glucose levels [109,110], while others do not confirm the effects of EGCG on amelioration of insulin resistance or glucose tolerance [111]. Even in healthy subjects the effect of green tea or EGCG consumption appear to be heterogeneous, with improved insulin sensitivity and glucose tolerance reported in some studies [112,113] and no relationship established in others [114].

These uncertainties may in part be explained by differences in bioavailability and biotransformation processes of EGCG among populations—or variations in the dose, the exposure time or the degree of pre-existing pathologic conditions. This suggests that future studies should take into account all these parameters, especially in the long-term administration, to ensure real benefits, avoid the risk of unwanted effects, and identify which type of intervention would constitute the most feasible and effective approach for preventing chronic metabolic derangements occurring in diabesity.

## 4. Effect of EGCG on Lipid Metabolism and Glucose Control

Beneficial effects on lipid metabolism and glucose homeostasis in response to EGCG have been consistently reported. Not surprisingly, mitochondrial function—including energy metabolism, biosynthesis and stress response—is improved by EGCG [115], whose in vivo administration in rats reduces oxidative damage, prevents the development of diet-induced obesity [39] and ameliorates peripheral insulin resistance induced by free FA (FFAs) infusion [116]. This ability of EGCG to limit insulin resistance correlates with the increased expression of antioxidant enzymes, including SOD and GPx, but also with phosphorylation of insulin receptor substrate-1 (IRS-1), activation of PKC and AMPK-dependent pathways, other kinases such as ERK1/2 and p38 MAPK, all essential for maximal stimulation of glucose uptake in response to insulin [116,117].

On animal model of diet-induced obesity, EGCG supplementation decreases body weight and adipose tissue mass and increases fatty acid oxidation via mRNA expression of UCP2 and UCP3 in liver and skeletal muscles [39,118]. Liver triglycerides and plasmatic levels of total cholesterol and glucose are also modulated by EGCG [119], that reduces body fat percentage, body weight gain and visceral fat with amelioration of metabolic syndrome and fatty liver disease [37]. For these effects, EGCG has been proposed to exert a modulatory activity on enzymes related to FA synthesis or FA oxidation: in obesity-prone mice, the dietary supplementation of EGCG reduces leptin expression in epididymal adipose tissue and expression of sterol–coenzyme A desaturase (SCD-1), malic enzyme and glucokinase in liver as well as in adipose tissue [39,42]. A downregulation of genes involved in gluconeogenesis and synthesis of FAs is also observed under EGCG administration in liver and adipose tissue of db/db mice, a genetic model of obesity and diabetes [99].

Molecular evidence supports the idea that EGCG elicits anti-obesity effects by regulating specific signaling pathways [87]. Activation of Sirtuin1 (SIRT1), a histone deacetylase involved in energy absorption and fat oxidation, has been proposed to explain some effects of EGCG [39]: SIRT1 is a NAD^+^-dependent deacetylase known to improve mitochondrial function and protect from metabolic disturbances by regulating the transcriptional co-factor peroxisome proliferator-activated receptor-γ–coactivator1α (PGC1α [120]. In mouse 3T3-L1 cells, as well in animals, EGCG mediates a concentration-dependent inhibition of adipocyte differentiation and viability and a decrease gluconeogenesis and fatty acid synthesis by activating AMP-activated protein kinase (AMPK) [121,122,123].

Interestingly, SIRT-1 and AMPK are two fuel-sensing molecules working in tandem and sharing common intracellular targets to regulate processes involved in mitochondrial biogenesis and function, cellular energy and metabolism, apoptosis and ROS reduction. As a master regulator of cellular metabolism in adipogenesis and fat oxidation, AMPK enzymatic activity stimulated by EGCG regulates homeostasis of FA and glucose metabolism in skeletal muscles and insulin secretion in pancreatic β-cells [124]. In rat hepatoma cells—as well as in isolated hepatocytes—EGCG downregulates genes controlling synthesis of FAs, triacylglycerol, cholesterol and gluconeogenesis and upregulates genes involved in glycogenesis, leading to reduced expression of gluconeogenic enzymes and decreased glucose production [125]. Thus, by AMPK-dependent signaling, EGCG increases the catabolism rate and decreases FA synthesis, resulting in body weight reduction and amelioration of metabolic syndrome [94,121,125]. Interestingly, EGCG-mediated AMPK activation is secondary to ROS generation, therefore underlying the importance of “pro-oxidant” activity of EGCG in its overall beneficial function.

Moreover, via activation of AMPK [126] and PI3K/Akt pathway [127,128], EGCG is able to upregulate the expression and the membrane translocation of GLUT4 in muscle cells in vitro and in animal model of diet-induced obesity [41,129] and to improve glucose tolerance and fasting blood glucose in an genetic model of obesity and type 2 diabetes [99].

## 5. Effect of EGCG on Endothelial Function

Under insulin resistance, as well as hyperglycemia and high circulating levels of FFAs, vascular tissues undergo profound changes that impair production of endogenous mediators and shift the balance toward a proinflammatory and pro-atherogenic phenotype. Inflammatory activation by ox-LDL uptake or by cytokines such as IL-1*β* and TNF-*α*, with subsequent increased expression of adhesion molecules, contributes to lipid accumulation within the atheroma and dysregulated activity of vascular smooth muscle cells [130,131,132,133].

In the vasculature, the major source of glucose-induced ROS production is a family of enzymes termed NADPH-oxidases [134,135,136,137,138], whose excessive activity has been directly related to cardiovascular complications of diabesity [16].

The earliest and most sensitive marker of vascular impairment is endothelial dysfunction, characterized by a reduced production of NO by the endothelial NO synthase (eNOS). Activity of eNOS is carefully regulated, among other mechanisms, by kinases such as AMPK and Akt and production of NO participates in maintenance of oxidative/reductive cell balance by limiting oxidative phosphorylation in mitochondria. In the presence of elevated ROS levels, eNOS is converted to a superoxide-producing enzyme by a mechanism termed eNOS uncoupling [139,140,141,142]. This, in turn, further aggravates oxidative stress by increasing the amounts of NO-derived oxidative species such as peroxinitrites, strong oxidants that alter structure and function of several cellular components [143,144].

EGCG is known to stimulate endothelial NO production via signaling pathways partially overlapping with insulin signaling, that require Fyn, PI3 kinase and Akt, to activate eNOS. [33,145]. Concomitantly, by activating AMPK signaling, EGCG decreases the expression of endothelin-1 (ET-1), reduces the NLRP3-mediated inflammation and apoptosis [146] and regulates the antioxidant status in endothelial cells [147], thus improving endothelial function with synergistic mechanisms that contribute to lower blood pressure, reduce insulin resistance, and protect against myocardial ischemia–reperfusion injury in animal models of metabolic syndrome [34,148]. Furthermore, EGCG protects against the endothelium barrier hyperpermeability induced by angiotensin (Ang) II via inactivation of p38 MAPK/HSP27 pathway [149], and prevents ectopic lipid accumulation in endothelial cells through a facilitated autophagic flux [47]. All these activities do not seem to be mediated by EGCG binding to cell surface receptors, since the presence of the laminin receptor (67LR), the putative membrane receptor for EGCG in cancerous cells, has not been demonstrated in endothelium [150,151,152].

However, EGCG has been shown to interact with lipid membranes, and modulate the topology, structure (hence function) of transmembrane receptors domains [153]. This may contribute to changes in angiogenic potential of endothelial cells, attributed to EGCG interaction with vascular endothelial growth factor receptor (VEGFR), as to the increased activity of the insulin receptor (InsR)-mediated signaling under EGCG treatment [154,155,156].

Thus, EGCG activates a number of intracellular signaling cascades in endothelium, under both physiological conditions and diabesity-related alterations. For several signaling pathways described above such as AMPK- and PI3K/Akt-mediated cascades, EGCG activation is triggered by generation of low levels of ROS [60,61,62]. For example, EGCG-stimulated production of ROS promotes activation of NF-E2-related factor 2 (Nrf2 transcription factor) and increases the expression of heme oxygenase 1 (HO-1) and glutathione in vascular endothelium. These effects serve to explain, at least in part, the EGCG-mediated protection from proinflammatory activities of TNF-α and contribute to overall cardiovascular beneficial effects of EGCG [157,158].

**Figure 2:**
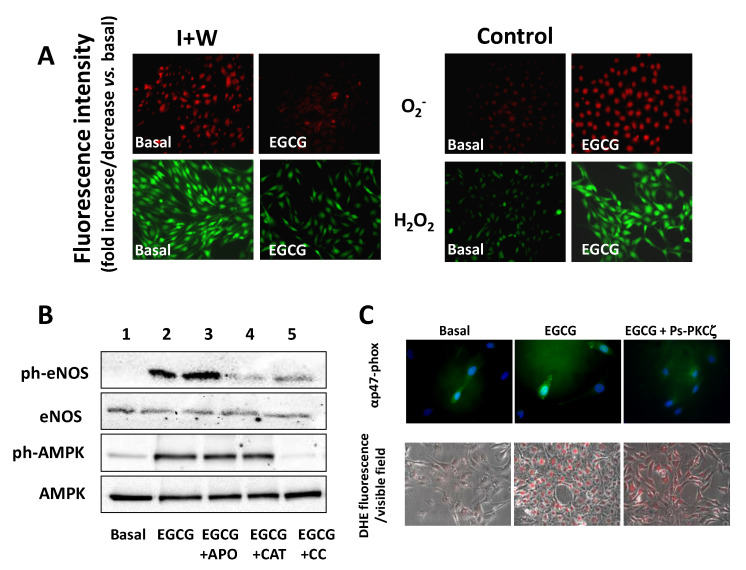
Effects of EGCG on ROS levels and signaling pathways related to endothelial NO synthase (eNOS) activation in endothelial cells. (**A**) EGCG decreases ROS overproduction in endothelial cells exposed to insulin resistance (high insulin levels, I + inhibition of PI 3 K wortmannin, W), but increases both H_2_O_2_ and O_2_^−^ in healthy endothelial cells (control). Red fluorescence indicates O_2_^−^; green fluorescence indicates H_2_O_2_; (**B**) EGCG increases phosphorylation of eNOS and AMPK in control BEC with a signaling that requires H_2_O_2_ and AMPK. Apocynin (APO) is a NADPH oxidase inhibitor; Catalase (CAT) is a H_2_O_2_ scavenger; Compound C (CC) is an AMPK inhibitor (**C**) EGCG increases the membrane localization of p47^phox^ in healthy endothelial cells via a PKCζ-mediated signaling. Green fluorescence indicates p47*^phox^*; blue fluorescence indicates cell nucleus; PS-PKCζ is an inhibitor of PKCζ.

While supporting the notion that ROS are important signaling molecules, these concepts reinforce the idea that EGCG may act as “antioxidant” or “pro-oxidant” molecule depending on the concentration employed and the pre-existing cell redox status. On this regard, we report some preliminary observations showing that the effects induced by increasing concentrations of EGCG (1 μM up to 50 μM/1 h) in bovine aortic endothelial cells (BEC) greatly differ under control conditions and under conditions mimicking hyperglycemia- and insulin resistance-mediated oxidative stress. While EGCG significantly decreases ROS overproduction in BEC exposed to high glucose or high insulin levels, EGCG dose-dependently increases both H_2_O_2_ and O_2_^−^ in healthy endothelial cells (Figure 2A).

Both these ROS species correlate with specific functions and act as triggering signals for EGCG-mediated effects in endothelial cells. H_2_O_2_ production is involved in eNOS activation in healthy conditions, confirming previous findings [33] and consistent with reduced EGCG-dependent eNOS phosphorylation when catalase was present in cell culture (Figure 2B). In this setting, EGCG involves AMPK and upstream CaMKKβ kinase to activate eNOS. CaMKKβ is a Ca-sensitive kinase ubiquitously expressed in endothelial cells [159,160] that participates to eNOS activation following Ca^2+^ release from endoplasmic reticulum (ER) stores and the ability of EGCG to stimulate Ca^2+^ release has been demonstrated in endothelium and other cell types [161]. Thus, the involvement of CaMKKβ supports the hypothesis that EGCG recruits signaling pathways involving calcium, CaMKKβ and AMPK, to activate eNOS in endothelial cells.

In response to higher doses, EGCG increases O_2_^−^ levels in healthy cells and abrogation of EGCG-mediated O_2_^−^ does not modify the ability of EGCG to enhance eNOS and AMPK phosphorylation (Figure 2B). On the other hand, high O_2_^−^ levels in response to elevated EGCG concentrations involve the atypical PKCζ isoform to facilitate the cell membrane localization of p47phox, a subunit of NADPH oxidase required to generate the active NADPH oxidase complex (Figure 2C). Binding of NADPH to a C-terminal site of a Nox enzyme allows the transfer of electrons to molecular oxygen to produce O_2_^−^ [162]. Therefore, this vicious circle may reinforce oxidative stress and translate into a self-sustaining mechanism that impairs endothelial cell function. Indeed, when control BEC were treated with the highest doses of EGCG, a higher susceptibility of cell detaching was observed, therefore supporting the negative impact of EGCG on cell viability and survival. These observations, indicating the ability of EGCG to modulate redox balance according the initial setting, may shed light on potential unwanted effects in an otherwise healthy cell environment and help explain the advantageous effects of EGCG-induced oxidative stress in a variety of cancerous cells [163].

**Figure 3:**
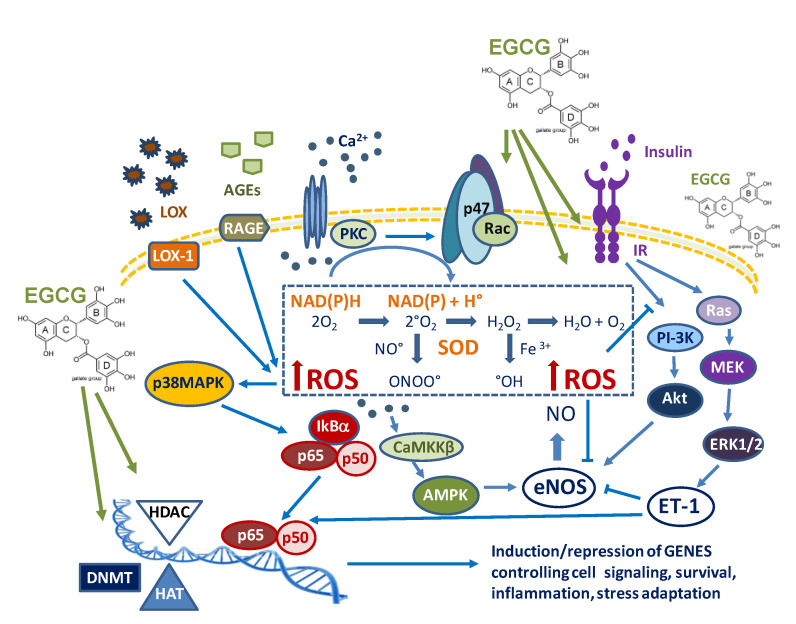
Schematic diagram showing main protective signaling mediated by EGCG in endothelial dysfunction under insulin resistance, lipotoxicity and glycotoxicity. EGCG modulates several signaling pathways, including the insulin receptor-dependent PI3K/Akt pathways and the Ras/MAPK pathways, the p38MAPK and the CaMKKβ/AMPK-mediated effects. At the nuclear level, EGCG also inhibits the DNA binding of effector transcription factors, such as NF-κB and modulates the induction/repression of specific genes by targeting DNA methylation (DNMT), histone acetylase (HAT) and histone deacetylase (HDAC) enzymes. These activities are suggested to involve, in part, an EGCG interference with cellular redox balance and, in part, a direct interaction of EGCG with the catalytic site of endogenous enzymes.

The main activities of EGCG in endothelial cells under diabesity are summarized in Figure 3.

Evolving knowledge on epigenetics of diabesity is expected to provide new critical information on progressive changes associated with vascular complications, as, for example, the role of metabolic memory on the pathogenesis of accelerated atherosclerosis or coronary artery disease [164].

Understanding the role played by oxidative stress and inflammation on regulation of gene expression in diabetes, obesity and insulin resistance has significantly increased attention toward EGCG effects, especially in the long-term administration, [165,166,167]. Along with mechanisms of transient regulation for protein function and transmitting signals, EGCG may contribute to DNA methylation, histone modifications, chromatin remodeling and expression/repression of non-coding RNAs.

Studies with structural analogs of EGCG underlie the importance of D and B ring and suggest that EGCG inhibits the enzyme DNA methyltransferase (DNMT) by direct interaction with the catalytic site. This also implies that EGCG is transported to the nucleus, where EGCG may reactivate methylation-induced gene silencing [168,169] and regulate chromatin structure by activity on histone acetyl transferase (HAT) and histone deacetylase (HDAC). These effects were linked to downregulated expression of genes controlled by NF-kB, such as inflammatory cytokines and adhesion molecules [170,171].

In the last years, the increasing understanding on the role and significance of microRNAs (miRNAs) has prompted investigation on potential additional effects of phytochemicals, including EGCG. MiRNAs are responsible for the fine-tuning of gene expression by controlling the expression of their target mRNAs in both normal and pathologic cells [164]. Noticeably, inflammation and ROS may induce the increase/decrease of several miRNA, including oxidative stress-responsive miRNAs. Obese, type 2 diabetic and diabesity patients show high levels of *c*irculating extracellular vesicles (EV), whose content may include, among others, miRN*A-*362-3p*,* -877-3p and -150-5p known to target mTOR substrates [172]. In parallel, members of the miRNA-29 family have been associated with impaired IRS-1, Akt and glycogen synthase kinase 3β (GSK3β) in both rat myocytes [173] and human pancreatic β-cells [174]. Upregulation of miR29a and miR30 has been found in peripheral blood mononuclear cells from patients with gestational diabetes, and seems involved in insulin signaling, angiogenesis, insulin resistance and adipose tissue dysfunction [175].

EGCG may exert its biologic function by up- or downregulation of multiple miRNA [176] and this effect seems to be related on exposure time. For example, a short incubation of EGCG (50 mg/L for five hours) down-regulates miR-30b expression in human hepatocellular carcinoma (HepG2) cells, while a longer incubation (100 μM for 24 h) reduces miR-181d and miR-222 expression in the same cells [176].

Although effects of EGCG on miRNA regulation are mostly documented in cancer diseases, recent studies have shown that treatment of obese mice with green tea extract for 12 weeks reduces the expression of miRNA-155 and miRNA-335. miRNA-335 is upregulated by TNFα in adipocytes and in turn, downregulates genes involved in insulin signaling and lipid metabolism [177]. These findings are consistent with the observation that EGCG, by upregulating miRNA-16, induces suppression of NF-kB activity and inhibition of M2 polarization of tumor-associated macrophages via IL-6, TGFβ and TNFα downregulation [178].

## 6. Conclusions

An impressive number of mechanisms and signaling pathways are proposed to explain the improved insulin sensitivity, lipid homeostasis, glucose uptake, endothelial protection and vascular performance following EGCG administration. Most of the signaling cascades involved in EGCG effects are known for their fundamental role on insulin signaling in metabolic and cardiovascular target tissues, therefore helping to explain the beneficial properties expected in patients with diabesity. Notwithstanding, findings from clinical studies not always agree with the effects produced by EGCG in vitro; a low bioavailability, a significant reduction in human plasma and/or body tissues for the concentrations used and potential differences according to the subpopulation genotype may contribute to current disappointing results.

However, findings suggesting a direct activity of EGCG on lipids and proteins on the surface of plasmatic membranes, as well as into intracellular compartments including mitochondria and nucleus, have broaden the spectrum of EGCG pleiotropic effects. These observations, together with the possibility that, with higher concentrations, the reducing power of EGCG may gradually predominate over its ROS scavenging activity, provide unquestionable evidence that EGCG displays a complex range of modulating effects beyond its traditional “antioxidant” characterization. The comprehensive description of signaling modulated by EGCG, together with the increasing recognition of its double-edge nature, may help to better evaluate the authentic advantages that could be obtained in patients with diabesity, and may undoubtedly represent a promising path for unconventional, non-pharmacological adjunctive approaches with a lower risk of toxicity.

## Abbreviations

AGEs	advanced glycosylated end products
AMPK	5’AMP-activated protein kinase
CaMKKβ	Ca/calmodulin-dependent protein kinase beta
DAG	diacylglycerol
DNMT	DNA methyltransferase
EGCG	epigallocatechin-3-gallate
eNOS	endothelial NO synthase
ERK	extracellular signal-regulated kinase
FFA	free fatty acids
GPx	glutathione peroxidase
HAT	histone acetyltransferase
HDAC	histone deacetylase
HSP27	heat shock protein 27
IRS-1	insulin receptor substrate-1
MAPK	mitogen-activated protein kinase
miRNA	microRNA
NF-kB	nuclear factor kappa-light-chain-enhancer of activated B cells
NLRP3	NLR family pyrin domain containing protein 3 inflammasome
NO	nitric oxide
NOX	NADPH oxidase enzyme
PGC1α	peroxisome proliferator-activated receptor-γ–coactivator1α
PI3K	phosphatidylinositol 3-kinase
PKC	protein kinase C
ROS	radical oxygen species
SCD-1	sterol-coenzyme A desaturase
SIRT-1	sirtuin-1
SOD	superoxide dismutase
T2DM	type 2 diabetes mellitus
TRXR	thioredoxin reductase
UCP2/3	uncoupling proteins 2 and 3
VEGF	vascular endothelial growth factor
